# Measles dynamics on network models with optimal control strategies

**DOI:** 10.1186/s13662-021-03306-y

**Published:** 2021-02-27

**Authors:** Yuyi Xue, Xiaoe Ruan, Yanni Xiao

**Affiliations:** School of Mathematics and Statistics, Xi’an Jiaotong University, Xi’an, P.R. China

**Keywords:** Measles, Network model, Basic reproduction number, Threshold dynamics, Optimal control

## Abstract

To investigate the influences of heterogeneity and waning immunity on measles transmission, we formulate a network model with periodic transmission rate, and theoretically examine the threshold dynamics. We numerically find that the waning of immunity can lead to an increase in the basic reproduction number $R_{0}$ and the density of infected individuals. Moreover, there exists a critical level for average degree above which $R_{0}$ increases quicker in the scale-free network than in the random network. To design the effective control strategies for the subpopulations with different activities, we examine the optimal control problem of the heterogeneous model. Numerical studies suggest us no matter what the network is, we should implement control measures as soon as possible once the outbreak takes off, and particularly, the subpopulation with high connectivity should require high intensity of interventions. However, with delayed initiation of controls, relatively strong control measures should be given to groups with medium degrees. Furthermore, the allocation of costs (or resources) should coincide with their contact patterns.

## Introduction

Measles, spread by coughing and sneezing, is one of the leading infectious diseases that causes death of children around the world. Vaccination is the key public health strategy to prevent it outbreaks. In 1986, China began to implement two doses of routine vaccination, at 8 months and 7 years old of age [1]. In 2010, a nationwide supplementary immunization activity (SIA) of measles was conducted in mainland China and subsequently the incidence of measles reached a low level of about $0.46/100\text{,}000$ individuals in 2012 [2]. However, in 2013, the incidence of measles rose again in mainland China. Then the repeated outbreaks occurred with a pattern that was small-scale and clustered. Until now, the elimination goal that the incidence of measles is less than $1/100\text{,}000$ population is still not achieved. Hence, how to design an effective strategy to eliminate the measles is still a challenge in mainland China.

Measles, like many other infectious diseases, exhibits great heterogeneity in terms of the number and the pattern of contacts. Much research indicated that heterogeneity may lead to highly different transmission dynamics of infectious diseases [3–8]. For example, the basic reproduction number, the threshold value to determine whether disease infection dies out or not, depends crucially on the variance of the distribution of contacts [9]. Accounting for the heterogeneity and non-random mixing, the meta-population basic reproduction numbers were about 70% greater [10]. Compared with the homogeneous mass-action model, the extinction probability of the disease before a large epidemic occurs is much lower for the network model [11]. These results mean that the structure of the contact network is crucial in investigating and characterizing transmission of epidemics. So, many researchers studied the spread of epidemics such as foot-and-mouth disease, SARS, dengue, A/H1N1, and AIDS [12–16] on complex networks and analyzed the threshold of outbreaks and stability of equilibriums [17–21], which can seize the epidemic dynamics and further devise control measures. However, few studies have cautioned on the importance of the contact structures in understanding measles spread. Hence, it is necessary to investigate the network structure and how it relates to the propagation of measles. Moreover, for measles, waning of immunity may be of great risk to induce more infections, since vaccinated individuals with sufficiently low antibody levels are either at risk of disease infection [22] or have newborn babies with low immunity, who have potential risk to be infected before 8 months. Hence, how the waning of immunity does affect the measles infection also falls within the scope of this study.

Furthermore, an important topic of epidemiology, control and elimination of infectious diseases, have been extensively investigated for measles [23–26]. Particularly, in 1957, Bartlett [27] found that the localized extinction of measles was related to the community size and then it was observed by Keeling and Grenfell [28] in 1997 that this threshold may depend on the spatial structure and connectedness of the population. In 1999, Roberts and Tobias [29] found that the second MMR (measles–mumps–rubella) immunization at or around school entry may offer logistic advantages in New Zealand. In 2015, Verguet and Johri [30] proposed that without second routine immunization, regular SIAs at high coverage are able to control measles transmission, but the periodicity of SIA campaigns is determined by population demographics and existing MCV1 (measles-containing vaccine) coverage. Moreover, Hao and Glasser [31] calculated the gradient of the effective number with respect to age- and location-specific immunization rates and obtained young adults are the optimal target for SIAs. It should be noted that the majority of this literature for control of measles is based on the homogeneous mixed assumption. Hence, under heterogeneous scenario, how to design effective control measures for different population to eliminate measles is another aim of this study.

Our purpose of this study is to propose a novel network model with periodic transmission by dividing the vaccinated individuals into two classes: some vaccinated individuals with high antibody titer who are completely protected, others with low antibody titer who may be infected. We investigate the effective control strategy in this high-dimension and heterogenous system by applying optimal control method. We define the basic reproduction number and theoretically analyze the threshold dynamics of the system. Then we examine the optimal control problem and numerically investigate the effect of network structure on the outbreak of measles. In particular, we combine three kinds of feasible control measures (self-protection, enhancement of vaccination and treatment) and apply optimal control theory to answer the question of how to design, with the variation of network structure, the optimal control measures for each subpopulation with different activities and how much each group cost.

## The network model

Each individual in a community can be regarded as a vertex in the network and each contact between two individuals is represented as an edge. The degree of a vertex means the number of contacts an individual has. In the following, the population is divided into *n* groups with sizes $N_{k}$ such that in group *k*, each individual has exactly *k* contacts for unit time. Assuming the population size is *N*, then the degree distribution of this network is defined as $p(k)=\frac{N_{k}}{N}$.

### Model formulation

In this model, each vertex is empty or occupied by at most one individual. New recruitment with rate of constant *b* can only happen at the empty vertex and an occupied vertex becomes empty after the individual dies with rate of *μ*. Let $S_{k}$, $V_{1k}$, $V_{2k}$, $E_{k}$, $E_{vk}$, $I_{k}$, $R_{k}$, $1-A_{k}$ be the densities of susceptible, vaccinated, exposed (infected from $S_{k}$ and $V_{2k}$), infected, recovered and empty nodes with degree *k*. The vaccinated individuals ($V_{1k}$) who are completely protected with high antibody titer progress to the class ($V_{2k}$) with waning of immunity at rate of *ω* and then can be infected because of low antibody titer. We can write

1 $$ \textstyle\begin{cases} \frac{dS_{k}}{dt}=b(1-A_{k})-\beta (t)kS_{k}\Theta -pS_{k}-\mu S_{k}, \\ \frac{dV_{1k}}{dt}=pS_{k}-\omega V_{1k}-\mu V_{1k}, \\ \frac{dV_{2k}}{dt}=\omega V_{1k}-\eta \beta (t)kV_{2k}\Theta -\mu V_{2k}, \\ \frac{dE_{k}}{dt}=\beta (t)kS_{k}\Theta -\sigma E_{k}-\mu E_{k}, \\ \frac{dE_{vk}}{dt}=\eta \beta (t)kV_{2k}\Theta -\sigma E_{vk}-\mu E_{vk}, \\ \frac{dI_{k}}{dt}=\sigma E_{k}+\sigma E_{vk}-\gamma I_{k}-\mu I_{k}, \\ \frac{dR_{k}}{dt}=\gamma I_{k}-\mu R_{k}, \end{cases} $$

where $A_{k}=S_{k}+V_{1k}+V_{2k}+E_{k}+E_{vk}+I_{k}+R_{k}$, and

$$ \Theta =\sum_{m} p(m|k)I_{m}= \frac{1}{\langle k \rangle } \sum_{m} mp(m)I_{m}, \quad {\langle k \rangle }=\sum_{k}kp(k). $$

**Figure 1:**
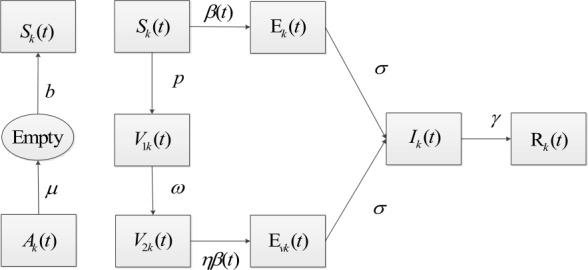
The flow diagram of the measles transmission on the network model ($A_{k}=S_{k}+V_{1k}+V_{2k}+E_{k}+E_{vk}+I_{k}+R_{k}$)

Here *p* is the vaccination rate of the susceptible individuals, $\frac{1}{\sigma }$ is the incubation period of measles, *γ* is the recover rate of the infected individuals, the periodic function $\beta (t)$ with period *T* is the baseline periodic transmission rate, *η* is the adjust factor which represents vaccinated individuals having reduced transmission rate ($0<\eta <1$). A flow diagram of the model is described in Fig. 1.

### The threshold dynamics

For the system (1), there exists an unique disease-free equilibrium $E_{0}=\{\bar{S}_{1},\bar{V}_{11},\bar{V}_{21}, 0,0,0, 0,\ldots ,\bar{S}_{M}, \bar{V}_{1M},\bar{V}_{2M},0,0,0,0\}$, where $\bar{S}_{k}=\frac{b\mu }{(b+\mu )(p+\mu )}$, $\bar{V}_{1k}= \frac{bp\omega }{(b+\mu )(p+\mu )(\omega +\mu )}$, $\bar{V}_{2k}=\frac{bp\mu }{(b+\mu )(p+\mu )(\omega +\mu )}$, $k=1, \ldots , M$. Here, *M* denotes the maximum degree of all nodes. Based on the method provided in [32], we define the basic reproduction number of the system (1) as follows. Let $y=(y_{1}, y_{2},\ldots , y_{3M}, y_{3M+1},\ldots , y_{7M})$ represent the state of nodes in each class with $y_{i}\geq 0$ and divide the compartments into two types: infected classes $(E_{k}, E_{vk},I_{k})$ relabeled by $i=1,\ldots , 3M$ and uninfected compartments $(S_{k},V_{1k},V_{2k},R_{k})$ relabeled by $i=3M+1,\ldots , 7M$. Then we write the system (1)

$$ \frac{dy_{i}}{dt}=g_{i}(t,y)=\mathcal{G}_{i}(t,y)- \mathcal{D}_{i}(t,y). $$

Denote

$$ G(t)=\biggl(\frac{\partial \mathcal{G}_{i}(t, E_{0})}{\partial y_{j}}\biggr)_{1 \leq i,j \leq 3M},\qquad D(t)=\biggl( \frac{\partial \mathcal{D}_{i}(t, E_{0})}{\partial y_{j}}\biggr)_{1\leq i,j \leq 3M}. $$

Specifically,

$$ G(t)= \begin{pmatrix} 0&0&G_{1}(t) \\ 0&0&G_{2}(t) \\ 0&0&0 \end{pmatrix},\qquad D(t)= \begin{pmatrix} D_{1}(t)&0&0 \\ 0&D_{2}(t)&0 \\ D_{3}(t)&D_{4}(t)&D_{5}(t) \end{pmatrix}, $$

where

$$\begin{aligned}& G_{1}(t)=\beta (t) \begin{pmatrix} \frac{\bar{S}_{1}p(1)}{\langle k \rangle }&\cdots &\frac{M\bar{S}_{1}p(M)}{\langle k \rangle } \\ \frac{2\bar{S}_{2}p(1)}{\langle k \rangle }&\cdots &\frac{2M \bar{S}_{2}p(M)}{\langle k \rangle } \\ \cdots &\cdots &\cdots \\ \frac{M\bar{S}_{M}p(1)}{\langle k \rangle }&\cdots &\frac{M^{2}\bar{S}_{M}p(M)}{\langle k \rangle } \end{pmatrix}, \\& G_{2}(t)=\eta \beta (t) \begin{pmatrix} \frac{\bar{V}_{21}p(1)}{\langle k \rangle }&\cdots &\frac{M \bar{V}_{21}p(M)}{\langle k \rangle } \\ \frac{2\bar{V}_{22}p(1)}{\langle k \rangle }&\cdots &\frac{2M\bar{V}_{22}p(M)}{\langle k \rangle } \\ \cdots &\cdots &\cdots \\ \frac{M\bar{V}_{2M}p(1)}{\langle k \rangle }&\cdots &\frac{M^{2}\bar{V}_{2M}p(M)}{\langle k \rangle } \end{pmatrix}, \end{aligned}$$

$D_{1}(t)=D_{2}(t)=diag(\sigma +\mu )$, $D_{3}(t)=D_{4}(t)=diag(-\sigma )$, $D_{5}(t)=diag(\gamma +\mu )$.

Let $Z(t,s)$, $t\geq s$, denote the evolution operator of the following system:

2 $$ \frac{dz}{dt}=-D(t)z. $$

Then $Z(t,s)$ satisfies

$$ \frac{d Z(t,s)}{dt}=-D(t)Z(t,s),\quad Z(s,s)=I, \forall t\geq s, $$

where *I* is the identity matrix and the monodromy matrix $\Psi _{-D}(t)$ of the system (2) is equal to $Z(t,0)$, $t\geq 0$.

Assume that $B_{T}$ is the ordered Banach space composed of all *T*-periodic functions from *R* to $R^{3M}$ with maximum norm $\|\cdot \|$ and then define a linear operator $L:B_{T} \rightarrow B_{T}$ as follows:

$$ (Lx) (t)= \int _{-\infty }^{t} Z(t,s)G(s)x(s) \,ds= \int _{0}^{\infty }Z(t,t-a)G(t-a)x(t-a)\,da. $$

Here, $x \in B_{T}$ denotes the initial distribution of infected individuals, $G(s)x(s)$ represents the distribution of those newly infected by the infectious persons who were introduced at time *s*. For $t\geq s$, $Z(t,s)G(s)x(s)$ is the distribution of infected individuals produced at time *s* and remain in this compartment at time *t*. We can define the basic reproduction number of the system (1) by the spectral radius of *L*, i.e.

$$ R_{0}=\rho (L). $$

For system (1), we can verify that the conditions (A1)–(A7) given in [32] hold and then derive the following theorem.

#### Lemma 1

*Assume that* (*A*1)*–*(*A*7) [32] *hold*, *then the following statements are valid*:

1. $R_{0}=1$ *if and only if* $\rho (\Psi _{G-D}(T))=1$.
2. $R_{0}<1$ *if and only if* $\rho (\Psi _{G-D}(T))<1$.
3. $R_{0}>1$ *if and only if* $\rho (\Psi _{G-D}(T))>1$.

#### Theorem 1

*The disease*-*free equilibrium* $E_{0}$ *is globally asymptotically stable if* $R_{0}<1$, *and unstable if* $R_{0}>1$.

#### Proof

Firstly, we illustrate that the disease-free equilibrium is locally stable. The Jacobian matrix of the system (1) at $E_{0}$ equals

$$ \tilde{J}(E_{0})= \begin{pmatrix} G(t)-D(t)&0 \\ J_{1}(t)&J_{2}(t) \end{pmatrix}, $$

where

$$\begin{aligned}& J_{1}(t)= \begin{pmatrix} \tilde{A}_{1}&\tilde{A}_{1}&\tilde{A}_{1}-G_{1}(t) \\ 0&0&0 \\ 0&0&\tilde{A}_{1}-G_{2}(t) \\ 0&0&\tilde{A}_{2} \end{pmatrix}, \qquad J_{2}(t)= \begin{pmatrix} \tilde{A}_{1}+\tilde{B}_{3}+\tilde{B}_{4}&\tilde{A}_{1}&\tilde{A}_{1}&\tilde{A}_{1} \\ \tilde{B}_{1}&\tilde{B}_{2}+\tilde{B}_{3}&0&0 \\ 0&-\tilde{B}_{2}&\tilde{B}_{3}&0 \\ 0&0&0&\tilde{B}_{3} \end{pmatrix}, \end{aligned}$$

with diagonal matrices $\tilde{A}_{1}$, $\tilde{A}_{2}$, $\tilde{B}_{1}$, $\tilde{B}_{2}$, $\tilde{B}_{3}$, whose elements are −*b*, *γ*, *p*, −*ω*, −*μ*, separately.

Clearly, all eigenvalues of $J_{2}$ have negative real parts. Then, combining with Lemma 1, that is, $\rho (\Psi _{G-D}(T))<1$ for $R_{0}<1$, we derive that the disease-free equilibrium $E_{0}$ is locally stable if $R_{0}<1$. Next, we will illustrate $E_{0}$ is globally attractive.

For the system (1), it satisfies

$$ \textstyle\begin{cases} \frac{dS_{k}}{dt}\leq b-b(S_{k}+V_{1k}+V_{2k})-(p+\mu ) S_{k}, \\ \frac{dV_{1k}}{dt}\leq pS_{k}-(\omega +\mu )V_{1k}, \\ \frac{dV_{2k}}{dt}\leq \omega V_{1k}-\mu V_{2k}. \end{cases} $$

Then, using the standard comparison theorem [33], we can prove that, for any $\epsilon >0$, there is $\tau _{1}>0$ such that $S_{k}(t)\leq \bar{S}_{k}+\epsilon $, $V_{1k}(t) \leq \bar{V}_{1k}+ \epsilon $, $V_{2k}(t) \leq \bar{V}_{2k}+\epsilon $ for $t>\tau _{1}$.

Furthermore, we introduce an auxiliary system

3 $$ \textstyle\begin{cases} \frac{d\hat{E}_{k}}{dt}= \beta (t)k(\bar{S}_{k}+\epsilon ) \hat{\Theta }-\sigma \hat{E}_{k}-\mu \hat{E}_{k}, \\ \frac{d\hat{E}_{vk}}{dt}= \eta \beta (t)k(\bar{V}_{2k}+\epsilon ) \hat{\Theta }-\sigma \hat{E}_{vk}-\mu \hat{E}_{vk}, \\ \frac{d\hat{I}_{k}}{dt}=\sigma \hat{E}_{k}+\sigma \hat{E}_{vk}- \gamma \hat{I}_{k}-\mu \hat{I}_{k}, \end{cases} $$

with $\hat{\Theta }=\sum_{m} p(m|k)\hat{I}_{m}$ and write it as

4 $$ \frac{d\hat{Z}}{dt}=\bigl(G(t)-D(t)+\epsilon Q(t)\bigr)\hat{Z}, $$

where $\hat{Z}=(\hat{E}_{1},\hat{E}_{v1},\hat{I}_{1},\ldots ,\hat{E}_{M}, \hat{E}_{vM},\hat{I}_{M})$ and

$$ Q(t)= \begin{pmatrix} 0&0&\tilde{G}_{1}(t) \\ 0&0&\eta \tilde{G}_{1}(t) \\ 0&0&0 \end{pmatrix},\qquad \tilde{G}_{1}(t)= \begin{pmatrix} \frac{\beta (t)}{\langle k \rangle }p(1)&\cdots &\frac{M \beta (t)}{\langle k \rangle }p(M) \\ \cdots &\cdots &\cdots \\ \frac{M\beta (t)}{\langle k \rangle }p(1)&\cdots &\frac{M^{2} \beta (t)}{\langle k \rangle }p(M) \end{pmatrix}. $$

If $R_{0}<1$, corresponding to $\rho (\Psi _{G-D}(T))<1$, we get $\rho (\Psi _{G-D+\epsilon Q}(T))<1 $ for enough small $\epsilon >0$ and consequently $\phi _{1}=\frac{1}{T}ln\rho (\Psi _{G-D+\epsilon Q}(T))<0$. By Lemma 2.1 of [34], it can be illustrated that there is a positive *T*-periodic function $c(t)=(c_{11}(t),c_{21}(t),c_{31}(t),\ldots ,c_{1M}(t), c_{2M}(t),c_{3M}(t))^{T}$ such that $e^{\phi _{1}t}c(t)$ is a solution of the system (4). We can choose a small value $\alpha _{1}>0$ and $\tau _{2}>\tau _{1}$ to satisfy $\hat{Z}(\tau _{2})\leq \alpha _{1} c(0)$, then the inequality $\hat{Z}(t)\leq \alpha _{1} c(t-\tau _{2})e^{\phi _{1}(t-\tau _{2})}$, $t \geq \tau _{2}$ holds.

Using the standard comparison theorem again, we get

$$ \bigl({E}_{1}(t),{E}_{v1}(t),{I}_{1}(t),\ldots ,{E}_{M}(t),{E}_{vM}(t),{I}_{M}(t)\bigr) \leq \hat{Z}(t)\leq \alpha _{1} c(t-\tau _{2})e^{\phi _{1}(t-\tau _{2})}, $$

which demonstrates $\lim_{t\rightarrow \infty } E_{k}(t)=0$, $\lim_{t \rightarrow \infty }E_{vk}(t)=0$, $\lim_{t\rightarrow \infty }I_{k}(t)=0$. Furthermore, considering the corresponding limit system

$$ \textstyle\begin{cases} \frac{dS_{k}}{dt}= b-b(S_{k}+V_{1k}+V_{2k})-(p+\mu ) S_{k}, \\ \frac{dV_{1k}}{dt}=pS_{k}-\omega V_{1k}-\mu V_{1k}, \\ \frac{dV_{2k}}{dt}= \omega V_{1k}-\mu V_{2k}, \end{cases} $$

we can derive $\lim_{t\rightarrow \infty } S_{k}(t)=\bar{S}_{k}$, $\lim_{t\rightarrow \infty }V_{1k}(t)=\bar{V}_{1k}$, $\lim_{t \rightarrow \infty }V_{2k}(t)=\bar{V}_{2k}$, $\lim_{t \rightarrow \infty }R_{k}(t)=0$. Above all, we have completely proved the global attractivity of $E_{0}$. □

#### Theorem 2

*If* $R_{0}>1$, *the system* (1) *is uniformly persistent and there exists at least a positive* *T*-*periodic solution*.

#### Proof

In the following, we first examine the uniform persistence of the system (1). This system admits a positive constant *ε̃* such that, for all initial values with $S^{0}_{k},V^{0}_{1k},V^{0}_{2k},R^{0}_{k}\geq 0$, $E^{0}_{k},E^{0}_{vk},I^{0}_{k}>0$, $k=1,\ldots , M$, the solution of system (1) satisfies $\lim_{t\rightarrow \infty } \inf I_{k}(t)\geq \tilde{\varepsilon }$, $\lim_{t\rightarrow \infty } \inf E_{k}(t) \geq \tilde{\varepsilon }$, $\lim_{t\rightarrow \infty } \inf E_{vk}(t) \geq \tilde{\varepsilon }$.

For simplicity, we let $Y_{k}=(S_{k},V_{1k},V_{2k},E_{k},E_{vk},I_{k},R_{k})$ and define $Y=\{(Y_{1}, Y_{2}\cdots ,Y_{M}):S_{k},V_{1k},V_{2k},E_{k},E_{vk},I_{k},R_{k} \geq 0,A_{k}\leq 1,k=1,\ldots ,M\}$, $Y_{0}=\{(Y_{1}, Y_{2}\cdots ,Y_{M}):S_{k},V_{1k},V_{2k},R_{k} \geq 0,E_{k},E_{vk},I_{k}> 0,A_{k}\leq 1,k=1,\ldots ,M\}$, $\partial Y_{0} =Y\backslash {Y_{0}}$. Obviously, *Y*, $Y_{0}$ are positively invariant and $\partial {Y_{0}}$ is relatively closed in *Y*.

Define Poincaré map $P:R_{+}^{7M}\rightarrow R_{+}^{7M}$ to satisfy $P(x_{0})=v(T,x_{0})$, $x_{0}\in R_{+}^{7M}$. $v(t,x_{0})$ is the solution of the system (1) with initial value $x_{0}$. Moreover, from the equations of system (1), we obtain $\lim_{t\rightarrow \infty } A_{k}(t)=\frac{b}{b+\mu }$, meaning that the solution of system (1) is uniformly and ultimately bounded. Hence, the semilow *P* is point dissipative and compact on $R_{+}^{7M}$. Theorem 3.4.8 of [35] indicates that the map *P* has a global attractor, which attracts each bounded set in $R_{+}^{7M}$.

Denote $Y_{\partial }=\{y_{0}=(Y_{1}^{0}, Y_{2}^{0},\ldots , Y_{M}^{0})\in \partial Y_{0}:P^{n}(y_{0})\in \partial Y_{0},\forall n>0\}$. Let $\tilde{Y}=\{(\tilde{Y}_{1},\tilde{Y}_{2}, \ldots ,\tilde{Y}_{M})\}$, where $\tilde{Y}_{k}=(S_{k},V_{1k},V_{2k},0,0,0,0)$, $k=1, \ldots ,M$. Next, we illustrate $Y_{\partial }=\tilde{Y}$. It is obvious that $\tilde{Y}\subseteq Y_{\partial }$. In the following, we only verify the relation $Y_{\partial }\subseteq \tilde{Y}$. Supposing that $y_{0}\in Y_{\partial }\backslash \tilde{Y}$, then a conflict occurs. For example, if $E_{1}^{0}>0$, $E_{k}^{0}=0$ ($k=2,\ldots , M$), $E_{vk}^{0}=I_{k}^{0}=0$ ($k=1,\ldots ,M$), then by the equations of the system (1), we deduce for sufficiently small $t >0$, $v(t,x_{0})\notin \partial Y_{0}$. This contradicts the initial assumption. Denote $\Omega =\bigcup \omega (y_{0})$, $y_{0}\in Y_{\partial }$. It is easy to see $\Omega =\{E_{0}\}$, that is, for any initial value from $Y_{\partial }$, the solution of system (1) will remain in $Y_{\partial }$.

To show $E_{0}$ is a weak repeller for $Y_{0}$, we only need to prove $W^{s}(E_{0})\cap Y_{0}=\emptyset $, where $W^{s}(E_{0})$ is the stable manifold of $E_{0}$. First, based on the continuity of solutions in terms of initial values, we see that $\forall \epsilon >0$, there is $\tilde{\delta }>0$ such that, for any $x_{0}\in Y_{0}$ which satisfy $d(x_{0},E_{0})\leq \tilde{\delta }$, $d(v(t,x_{0}),v(t,E_{0}))\leq \epsilon $ holds for $t \in [0,T]$. In the following, we demonstrate $\lim_{n\rightarrow \infty } \sup d(P^{n}(x_{0}),E_{0})\geq \tilde{\delta }$.

Suppose not, there is some $x_{0}\in Y_{0}$ such that $\lim_{n\rightarrow \infty }\sup d(P^{n}(x_{0}),E_{0})< \tilde{\delta }$. Without loss of generality, we assume for any $n>0$, $d(P^{n}(x_{0}),E_{0})<\tilde{\delta }$. Given $t>0$, let $t=nT+\tau _{3}$, then $d(v(t,x_{0}),v(t,E_{0}))=d(v(\tau _{3},P^{n}(x_{0})), v(\tau _{3},E_{0}))< \epsilon $. Let $(S_{k}(t),V_{1k}(t),V_{2k}(t),E_{k}(t),E_{vk}(t),I_{k}(t), R_{k}(t))=v(t,x_{0})$. The result indicates $\|S_{k}(t)-\bar{S}_{k}\|\leq \epsilon $, $\|V_{1k}(t)-\bar{V}_{1k}\|\leq \epsilon $, $\|V_{2k}(t)-\bar{V}_{2k}\|\leq \epsilon $, $\|E_{k}(t)\|\leq \epsilon $, $\|E_{vk}(t)\|\leq \epsilon $, $\|I_{k}(t)\|\leq \epsilon $, $k=1, \ldots ,M$. Then the fourth and fifth equations of the system (1) satisfy

$$ \textstyle\begin{cases} \frac{dE_{k}}{dt}\geq \beta (t)k (\bar{S}_{k}-\epsilon )\Theta -( \sigma +\mu )E_{k}, \\ \frac{dE_{vk}}{dt}\geq \eta \beta (t)k(\bar{V}_{2k}-\epsilon )\Theta -( \sigma +\mu )E_{vk}. \end{cases} $$

Introducing the auxiliary system

5 $$ \textstyle\begin{cases} \frac{d\tilde{E}_{k}}{dt}= \beta (t)k (\bar{S}_{k}-\epsilon ) \tilde{\Theta }-(\sigma +\mu )\tilde{E}_{k}, \\ \frac{d\tilde{E}_{vk}}{dt}= \eta \beta (t)k(\bar{V}_{2k}-\epsilon ) \tilde{\Theta }-(\sigma +\mu )\tilde{E}_{vk}, \\ \frac{d\tilde{I}_{k}}{dt}= \sigma (\tilde{E}_{k}+\tilde{E}_{vk})-( \gamma +\mu )\tilde{I}_{k}, \end{cases} $$

with $\tilde{\Theta }=\sum_{m} p(m|k)\tilde{I}_{m}$ and for convenience, we rewrite (5) as

$$ \frac{d\tilde{Z}}{dt}=\bigl(G(t)-D(t)-\epsilon Q(t)\bigr)\tilde{Z}, $$

where $\tilde{Z}=(\tilde{E}_{1}(t),\tilde{E}_{v1}(t),\tilde{I}_{1}(t), \ldots ,\tilde{E}_{M}(t),\tilde{E}_{vM}(t),\tilde{I}_{M}(t))$ and $G(t)$, $D(t)$, $Q(t)$ have the same form as mentioned in Theorem 1.

Following Lemma 2.1 [34], we can get a positive *T*-periodic function $\tilde{c}(t)=(\tilde{c}_{11}(t),\tilde{c}_{21}(t), \tilde{c}_{31}(t), \ldots ,\tilde{c}_{1M}(t),\tilde{c}_{2M}(t),\tilde{c}_{3M}(t))^{T}$ such that $e^{\phi _{2}t}\tilde{c}(t)$ is a solution of the system (5) with $\phi _{2}=\frac{1}{T}\ln \rho (\Psi _{G-D-\epsilon Q}(T))$. For $R_{0}>1$ (i.e. $\rho (\Psi _{G-D}(T))>1$), there exists a enough small $\epsilon >0$ such that $\rho (\Psi _{G-D-\epsilon Q}(T))>1$, which corresponds to $\phi _{2}>0$. Choosing $\tilde{\alpha }_{1}>0$, $t_{0}>0$ such that $\tilde{Z}(t_{0})\geq \tilde{\alpha }_{1} \tilde{c}(0)$, then the inequality $\tilde{Z}(t)\geq \tilde{\alpha }_{1} \tilde{c}(t-t_{0})e^{\phi _{2}(t-t_{0})}$ holds. Furthermore, the standard comparison theorem [33] demonstrates that the following inequality is established:

$$ \bigl(E_{1}(t),E_{v1}(t),I_{1}(t),\ldots ,E_{M}(t),E_{vM}(t),I_{M}(t)\bigr) \geq \tilde{Z}(t)\geq \tilde{\alpha }_{1} \tilde{c}(t-t_{0})e^{\phi _{2}(t-t_{0})}. $$

This indicates $\lim_{m\rightarrow \infty }E_{k}(t)=+\infty $, $\lim_{m \rightarrow \infty }E_{vk}(t)=+\infty $, $\lim_{m\rightarrow \infty }I_{k}(t)=+\infty $, which contradicts with $E_{k}(t),E_{vk}(t),I_{k}(t)<\epsilon $. Thus, we obtain $W^{s}(E_{0})\cap X_{0}=\emptyset $.

Above all, based on the Theorem 3.11 of [36], it is proved that the Poincaré map *P* is uniformly persistent with respect to $(Y_{0},\partial Y_{0})$. Furthermore, combining Theorem 1.3.6 [36], it can be proved that the Poincaré map has a fixed point $E^{*}=(S_{1}^{*},V_{11}^{*},V_{21}^{*},E_{1}^{*},E_{v1}^{*},I_{1}^{*},R_{1}^{*}, \ldots , S_{M}^{*},V_{1M}^{*},V_{2M}^{*},E_{M}^{*},E_{vM}^{*},I_{M}^{*}, R_{M}^{*})\in Int(R_{+}^{7M})$, hence we can derive that the solution $v(t,E^{*})$ is a positive and *T*-periodic solution of the system (1). This completes the proof. □

### Numerical study

**Figure 2:**
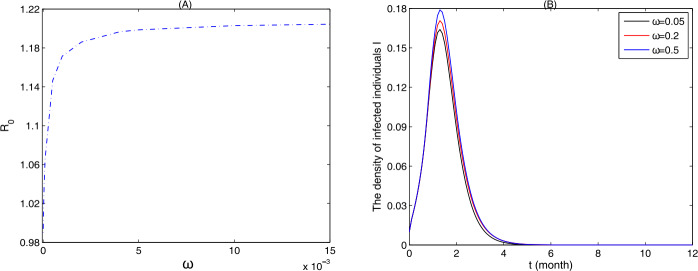
(**A**) The variation in $R_{0}$ with respect to the waning rate *ω* of immunity on the random network; (**B**) the density of infected individuals ($I=\sum_{k=1}^{M}I_{k}(t)$) on the whole random network with $\omega =0.05, 0.2, 0.5$

In this section, we initially analyze the effects of waning immunity on the transmission of measles in the random network and scale-free (SF) network. For the random network, it follows from Fig. 2(A) that increasing the waning rate (*ω*) of immunity can result in a significant increase in the basic reproduction number $R_{0}$, indicating that waning of immunity brings about a difficulty in eliminating the measles. And it is worth noting that, given there is no waning of immunity, like in many existing models, one may underestimate the value of $R_{0}$. Furthermore, Fig. 2(B) shows that an increasing waning rate leads to an increase in the density of infected nodes, especially the peak magnitude. This implies the waning of immunity causes many new infections and suggests us that prolonging the protection duration of vaccine is beneficial to control transmission of measles. Note that the plots for the SF network are similar to Fig. 2, and hence we omit them.

The basic reproduction number $R_{0}$ is the threshold to determine whether the measles dies out or not, hence it is essential to study the influence of the network structure determined by the degree distribution on $R_{0}$. Here, we continue to discuss a scale-free (SF) network and a random network. In the SF network, the degree distribution follows the power-law distribution (that is, $p(k)=mk^{-v}$, where *m* is uniquely determined by *v*) [37]. In the random network, the node degree follows the Poisson distribution (that is, $p(k)=\frac{K_{a}^{k}e^{-K_{a}}}{k!}$, the average degree $K_{a}=Nq$, where *N* and *q* represent the network size and the probability of connection between two nodes) [38].

**Figure 3:**
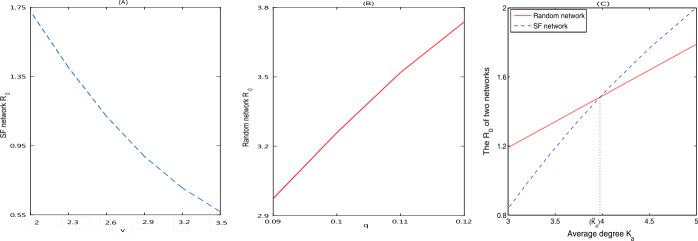
(**A**)–(**B**) The variation in $R_{0}$ with respect to *v* in the SF network and *q* in the random network; (**C**) the comparison of $R_{0}$ under different networks (SF network and random network) with the same average degree. $\bar{K}_{a}$ is a critical value of average degree

To study the impact of degree distribution on the basic reproduction number $R_{0}$, we examine the variation in $R_{0}$ with parameter *v* (SF network) and *q* (random network) changing. Figure 3(A) shows that as the parameter *v* in power-law distribution increases, the proportion of nodes with large degree decreases, causing a reduction in $R_{0}$. Hence, in the SF network, treating and isolating the infected individuals who have more connections to others can effectively control the spread of disease. Figure 3(B) indicates that in a random network, decreasing the probability *q* of random contacts between nodes can reduce the new infections.

To further explore the influence of network structure on the outbreak of disease, we compare the basic reproduction number $R_{0}$ between the SF network and the random network for a given average degree. Figure 3(C) indicates that there is a critical level $\bar{K}_{a}$ for the average degree (here $\bar{K}_{a}=3.9$) below which the basic reproduction number $R_{0}$ for the random network is greater than those for the SF network, above which the opposite result is observed. This means that the contact structure being like random network promotes the outbreak of measles when the average degree of nodes is small, while as the average degree increases, the proportion of nodes with large degree also increases and then measles can transmit faster in the SF network than in the random network.

## Optimal control strategies

In this section, we consider some feasible interventions and then extend the system (1) to the following model with control functions $u_{1k}(t)$, $u_{2k}(t)$, $u_{3k}(t)$:

6 $$ \textstyle\begin{cases} \frac{dS_{k}}{dt}=b(1-A_{k})-(1-u_{1k}(t))\beta (t)kS_{k}\Theta -p(1+u_{2k}(t))S_{k}- \mu S_{k}, \\ \frac{dV_{1k}}{dt}=p(1+u_{2k}(t))S_{k}-\omega V_{1k}-\mu V_{1k}, \\ \frac{dV_{2k}}{dt}=\omega V_{1k}-\eta (1-u_{1k}(t))\beta (t)kV_{2k} \Theta -\mu V_{2k}, \\ \frac{dE_{k}}{dt}=(1-u_{1k}(t))\beta (t)kS_{k}\Theta -\sigma E_{k}- \mu E_{k}, \\ \frac{dE_{vk}}{dt}=\eta (1-u_{1k}(t))\beta (t)kV_{2k}\Theta -\sigma E_{vk}- \mu E_{vk}, \\ \frac{dI_{k}}{dt}=\sigma E_{k}+\sigma E_{vk}-(1+u_{3k}(t))\gamma I_{k}- \mu I_{k}, \\ \frac{dR_{k}}{dt}=(1+u_{3k}(t))\gamma I_{k}-\mu R_{k}, \end{cases} $$

where $\Theta =\sum_{m=1}^{M} p(m|k)I_{m}= \frac{1}{\langle k \rangle }\sum_{m=1}^{M} mp(m)I_{m}$, ${ \langle k \rangle }=\sum_{k=1}^{M}kp(k)$. The control function $u_{1k}(t)$ represents the reduction in contacts of susceptible individuals with degree *k* by personal protection or social distancing measures. The control functions $u_{2k}(t)$, $u_{3k}(t)$ denote the enhanced vaccination and treatment in the group with degree *k*. For minimizing the infected individuals in the time interval $[0,T]$ with minimal cost, we define the objective function

$$ O(u_{1k},u_{2k},u_{3k})=\sum _{k=1}^{M} \int _{0}^{T} \biggl(N_{k}I_{k}(t)+ \frac{1}{2}W_{1k}u_{1k}^{2}(t)+ \frac{1}{2}W_{2k}u_{2k}^{2}(t)+ \frac{1}{2}W_{3k}u_{3k}^{2}(t)\biggr)\,dt, $$

where $W_{1k}=W_{1}N_{k}$, $W_{2k}=W_{2}N_{k}$, $W_{3k}=W_{3}N_{k} $ are the weights in terms of the control functions $u_{1k}$, $u_{2k}$, $u_{3k}$ with positive constants $W_{i}$, $i=1, 2,3$, $k=1,\ldots ,M$. We can derive the optimal control strategies by searching for the optimal control function $U^{*}(t)$ subject to the system (6) such that

$$ O\bigl(U^{*}(t)\bigr)=\min_{\tilde{\Omega }}{O}\bigl(U(t) \bigr), $$

where $\tilde{\Omega }=\{U(t)=(u_{11}(t),u_{21}(t),u_{31}(t),\ldots ,u_{1M}(t),u_{2M}(t),u_{3M}(t)) \in L^{2}(0,T)^{3M}| 0\leq u_{1k}(t)\leq \hat{u}_{1k},0\leq u_{2k}(t)\leq \hat{u}_{2k},0 \leq u_{3k}(t)\leq \hat{u}_{3k},k=1,\ldots , M\}$ with positive upper bounds $\hat{u}_{ik}$, $i=1,2,3$.

### Optimal control and optimal solutions

Since the state system is a linear function for *U* and satisfies the Lipschitz property for the state variables, while the integrand of *O* is a convex function in terms of $(u_{11}(t),u_{21}(t),u_{31}(t),\ldots ,u_{1M}(t),u_{2M}(t),u_{3M}(t))$, we can easily prove the existence of optimal controls by Corollary 4.1 of Fleming’s reference [39] and derive the following result.

#### Theorem 3

*There are the optimal control function* $U^{*}(t)$ *and the corresponding optimal solution* $C^{*}(t)$ *to minimize* $O(U(t))$ *over the* Ω̃. *In order to make the above statement correct*, *it is necessary to have continuous functions* $\lambda _{ik}(t)$ *such that*

7 $$\begin{aligned} \textstyle\begin{cases} \lambda _{1k}'(t)=\lambda _{1k}b+(\lambda _{1k}-\lambda _{4k})(1-u_{1k}(t)) \beta (t)k\Theta +(\lambda _{1k}-\lambda _{2k})p(1+u_{2k}(t)) \\ \hphantom{\lambda _{1k}'(t)=}{}+\lambda _{1k}\mu , \\ \lambda _{2k}'(t)=\lambda _{1k}b+(\lambda _{2k}-\lambda _{3k})\omega + \lambda _{2k}\mu , \\ \lambda _{3k}'(t)=\lambda _{1k}b+(\lambda _{3k}-\lambda _{5k})\eta (1-u_{1k}(t)) \beta (t)k\Theta +\lambda _{3k}\mu , \\ \lambda _{4k}'(t)=\lambda _{1k}b+(\lambda _{4k}-\lambda _{6k})\sigma + \lambda _{4k}\mu , \\ \lambda _{5k}'(t)=\lambda _{1k}b+(\lambda _{5k}-\lambda _{6k})\sigma + \lambda _{5k}\mu , \\ \lambda _{6k}'(t)=-N_{k}+\lambda _{1k}b+(\lambda _{1k}-\lambda _{4k})(1-u_{1k}(t)) \frac{\beta (t)k^{2}p(k)S_{k}}{\langle k\rangle }+(\lambda _{3k}- \lambda _{5k}) \\ \hphantom{\lambda _{6k}'(t)=}{}\times (1-u_{1k}(t)) \frac{\eta \beta (t)k^{2}p(k)V_{2k}}{\langle k\rangle } +(\lambda _{6k}- \lambda _{7k})(1+u_{3k}(t))\gamma +\lambda _{6k}\mu , \\ \lambda _{7k}'(t)=\lambda _{1k}b+\lambda _{7k}\mu , \end{cases}\displaystyle \end{aligned}$$

*with the transversality conditions*,

$$ \lambda _{1k}(T)=\lambda _{2k}(T)=\cdots =\lambda _{7k}(T)=0. $$

*Furthermore*, *the optimal controls are*

8 $$ \begin{gathered} u_{1k}^{*}(t)=min\biggl\{ max \biggl\{ \frac{(\lambda _{4k}-\lambda _{1k})\beta (t)kS_{k}^{*}(t)\Theta ^{*}(t)+(\lambda _{5k}-\lambda _{3k})\eta \beta (t)kV_{2k}^{*}(t)\Theta ^{*}(t)}{W_{1k}},0 \biggr\} ,\\ \hphantom{ u_{1k}^{*}(t)=} \hat{u}_{1k}\biggr\} , \\ u_{2k}^{*}(t)=min\biggl\{ max\biggl\{ \frac{(\lambda _{1k}-\lambda _{2k})p S_{k}^{*}(t)}{W_{2k}},0 \biggr\} , \hat{u}_{2k}\biggr\} , \\ u_{3k}^{*}(t)=min\biggl\{ max\biggl\{ \frac{(\lambda _{6k}-\lambda _{7k})\gamma I_{k}^{*}(t)}{W_{3k}},0 \biggr\} , \hat{u}_{3k}\biggr\} ,\quad k=1,2,\ldots ,M. \end{gathered} $$

#### Proof

Based on the Pontryagin’s maximum principles [40], we can get the necessary conditions satisfied by the optimal controls. Specifically, we first define the Hamilton function *H* as

$$ \begin{aligned} H&=\sum_{k=1}^{M} \biggl(N_{k}I_{k}+\frac{1}{2}W_{1k}u_{1k}^{2}+ \frac{1}{2}W_{2k}u_{2k}^{2}+ \frac{1}{2}W_{3k}u_{3k}^{2}+\lambda _{1k} \frac{dS_{k}}{dt}+\lambda _{2k}\frac{dV_{1k}}{dt} \\ &\quad {}+\lambda _{3k}\frac{dV_{2k}}{dt} +\lambda _{4k} \frac{dE_{k}}{dt}+ \lambda _{5k}\frac{dE_{vk}}{dt} +\lambda _{6k}\frac{dI_{k}}{dt}+ \lambda _{7k}\frac{dR_{k}}{dt} \biggr). \end{aligned} $$

The corresponding adjoint equations with transversality conditions are $\lambda '_{1k}=-\frac{\partial H}{\partial S_{k}}$, $\lambda '_{2k}=- \frac{\partial H}{\partial V_{1k}}$, $\lambda '_{3k}=- \frac{\partial H}{\partial V_{2k}}$, $\lambda '_{4k}=- \frac{\partial H}{\partial E_{k}}$, $\lambda '_{5k}=- \frac{\partial H}{\partial E_{vk}}$, $\lambda '_{6k}=- \frac{\partial H}{\partial I_{k}}$, $\lambda '_{7k}=- \frac{\partial H}{\partial R_{k}} $, with $\lambda _{1k}(T)= \lambda _{2k}(T)= \lambda _{3k}(T)= \lambda _{4k}(T)= \lambda _{5k}(T)= \lambda _{6k}(T)= \lambda _{7k}(T)=0$.

Differentiating *H* concerning $u_{1k}$, $u_{2k}$, $u_{3k}$ on the set Ω̃, respectively, the Hamilton function *H* can reach the minimum with the following optimal conditions:

$$ \frac{\partial H}{\partial u_{1k}}=0,\qquad \frac{\partial H}{\partial u_{2k}}=0, \qquad \frac{\partial H}{\partial u_{3k}}=0. $$

Solving for $u_{1k}^{*}(t)$, $u_{2k}^{*}(t)$, $u_{3k}^{*}(t)$, we get

$$ \begin{aligned} &u_{1k}^{*}(t)= \frac{(\lambda _{4k}-\lambda _{1k})\beta (t)kS_{k}^{*}(t)\Theta ^{*}(t)+(\lambda _{5k}-\lambda _{3k})\eta \beta (t)kV_{2k}^{*}(t)\Theta ^{*}(t)}{W_{1k}}, \\ &u_{2k}^{*}(t)= \frac{(\lambda _{1k}-\lambda _{2k})p S_{k}^{*}(t)}{{W_{2k}}},\qquad u_{3k}^{*}(t)= \frac{(\lambda _{6k}-\lambda _{7k})\gamma I_{k}^{*}(t)}{{W_{3k}}}. \end{aligned} $$

Combining with the bounds $0\leq u_{1k}(t)\leq \hat{u}_{1k}$, $0\leq u_{2k} \leq \hat{u}_{2k}$, $0\leq u_{2k}\leq \hat{u}_{3k}$, $k=1,\ldots ,M$, we derive the properties as Theorem 3. □

### Numerical simulation

In the following simulations, we mainly discuss the optimal control strategies and their corresponding effects on the incidence under two kinds of networks: random network and SF network. Here, we assume the community size $N=300$ and the maximum degree $M=21$.

#### Optimal control strategy on random network

**Figure 4:**
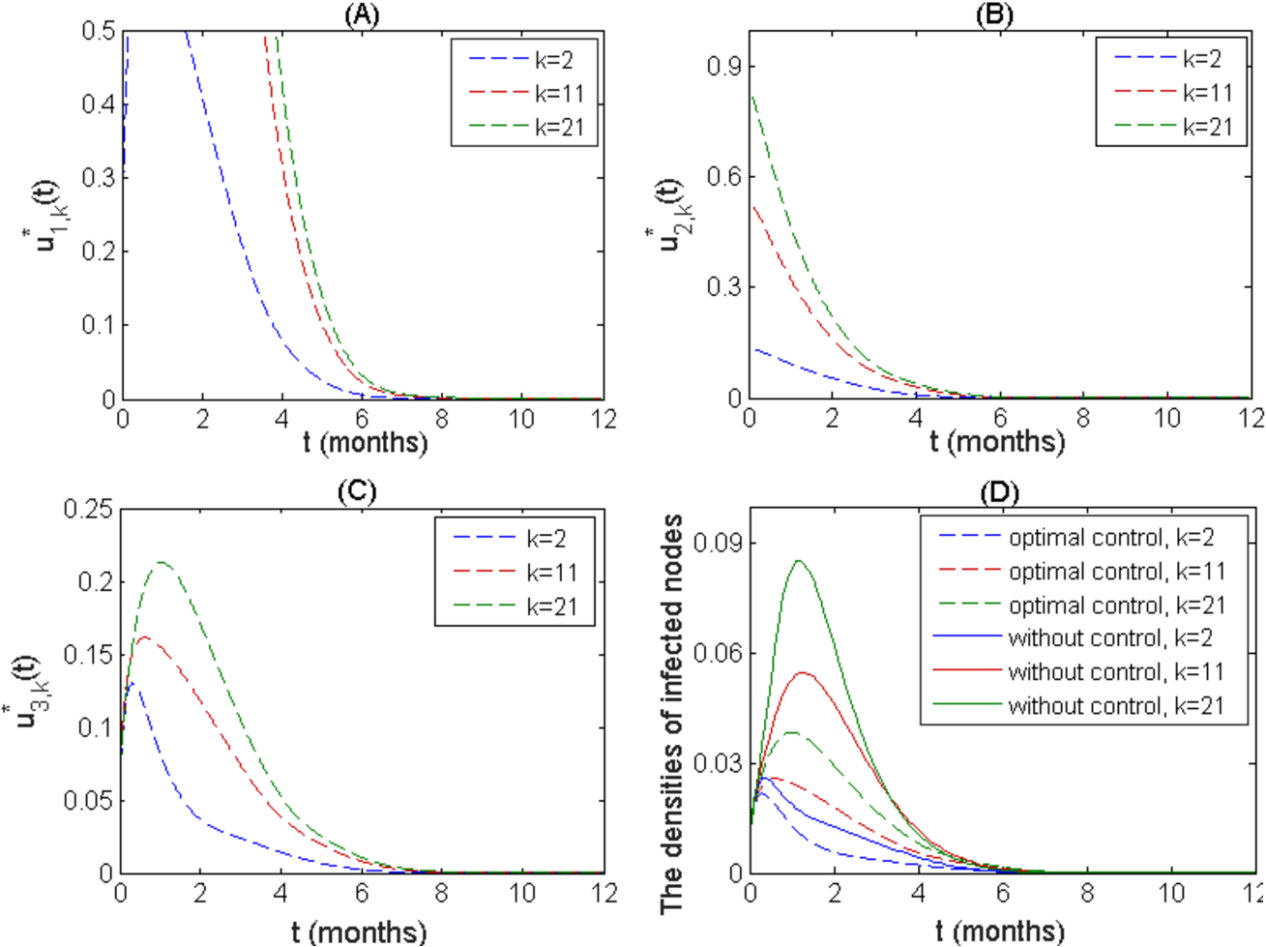
(**A**)–(**C**) The time series of optimal control functions for 3 groups with degree 2,11,21; (**D**) the densities of infected nodes for these 3 groups in the absence of control (solid line) and under optimal control (dotted line)

To study the effect of optimal control strategies on the transmission of measles under different groups, we plot the time series of optimal control functions and the corresponding densities of infected nodes with degree $k=2, 11,21$, respectively. It is observed from Fig. 4(A)–(C) that the optimal control function $u^{*}_{1k}(t)$ begins to decline after a period of plateau with the highest level, and $u^{*}_{2k}(t)$ monotonically decreases while the optimal control function $u^{*}_{3k}(t)$ initially increases and then decreases which behaves like infection incidence. This demonstrates that we should initiate personal protection and enhance vaccination as soon as possible when disease takes off while the implementation of treatment measures should be in sync with transmission of measles. Moreover, the optimal control curves show that the nodes with high degrees require relatively long duration of self protection with maximum level and strong initial vaccination strength to minimize the objective function. Figure 4(D) indicates that with optimal controls, the outbreak can be significantly mitigated. Furthermore, the larger the degree *k* is, the higher the peak value of $I_{k}(t)$ is, which means the population with high degree is easily and seriously invaded by disease, so we should pay more attention to the individuals in these groups.

To further explore what interventions should be taken for different groups and their effectiveness on disease spread, we define the average control strength

$$ \bar{u}_{ik}=\frac{1}{T} \int _{0}^{T} u^{*}_{ik}(t)\,dt, \quad i=1,2,3, $$

under the optimal controls and compare the cumulative densities of infected individuals $\bar{I}_{k}$, $\bar{I}^{*}_{k}$ over a period under two cases: without/with optimal control, where

$$ \bar{I}_{k}= \int _{0}^{T} I_{k}(t)\,dt, \qquad \bar{I}^{*}_{k}= \int _{0}^{T}I^{*}_{k}(t)\,dt, \quad k=1, 2, \ldots , M. $$

**Figure 5:**
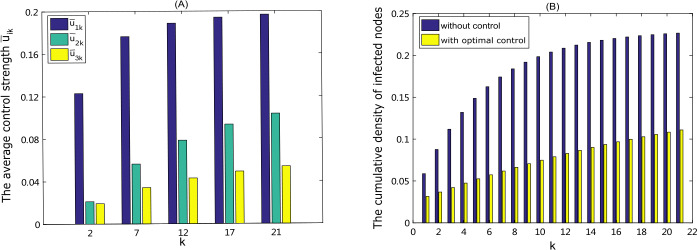
(**A**) Under the optimal controls, the average control strength $\bar{u}_{1k}$, $\bar{u}_{2k}$, $\bar{u}_{3k}$ for different groups $k=2,7,12,17,21$; (**B**) the cumulative densities of infected nodes for each group with/without optimal control

It follows from Fig. 4 (A) that the average control strength $\bar{u}_{ik}$ increases with the degree *k* increasing. This indicates that the control measures related to the highly connected individuals should be strengthened to achieve optimal control. And further, for a given degree *k*, we have $\bar{u}_{1k}>\bar{u}_{2k}>\bar{u}_{3k}$, which means that the control strategies such as personal protection and social distance, associated with reducing transmission, should be significantly implemented for each group. Figure 5(B) demonstrates that the implementation of optimal controls yields a reduction of more than 50% in the cumulative density of infected individuals for each group.

**Figure 6:**
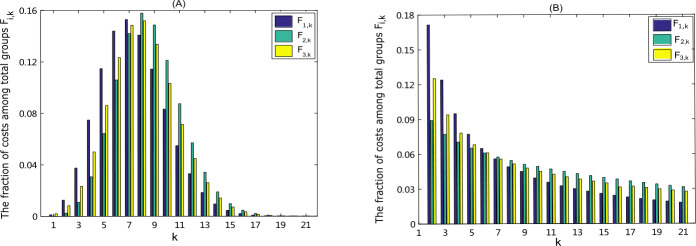
The distribution of each control costs among total groups in the random network (**A**) and SF network (**B**), which is illustrated by the fraction $F_{i,k}=\int _{0}^{T} W_{ik}u^{*}_{ik}(t)\,dt/\sum_{k=1}^{M} \int _{0}^{T} W_{ik}u^{*}_{ik}(t)\,dt$, $i=1,2,3$, $k=1,\ldots ,M$

To investigate the allocation of costs (or resources) in different groups under the optimal controls, we compute the fraction $F_{i,k}$ of costs occupied by each group for each strategy, as shown in Fig. 6(A), where

$$ F_{i,k}= \int _{0}^{T} W_{ik}u^{*}_{ik}(t)\,dt\Big/ \sum_{k=1}^{M} \int _{0}^{T} W_{ik}u^{*}_{ik}(t)\,dt, \quad i=1,2,3, k=1,\ldots , M. $$

It displays that the cost distribution among different groups represents the shape of normal distribution and a substantially large fraction of the costs is occupied by the nodes with medium degrees, indicating that when the measles spreads among individuals whose contact structure is more like random network, more resources such as facial masks, vaccines, medicines should be allocated to the population with medium degrees.

**Figure 7:**
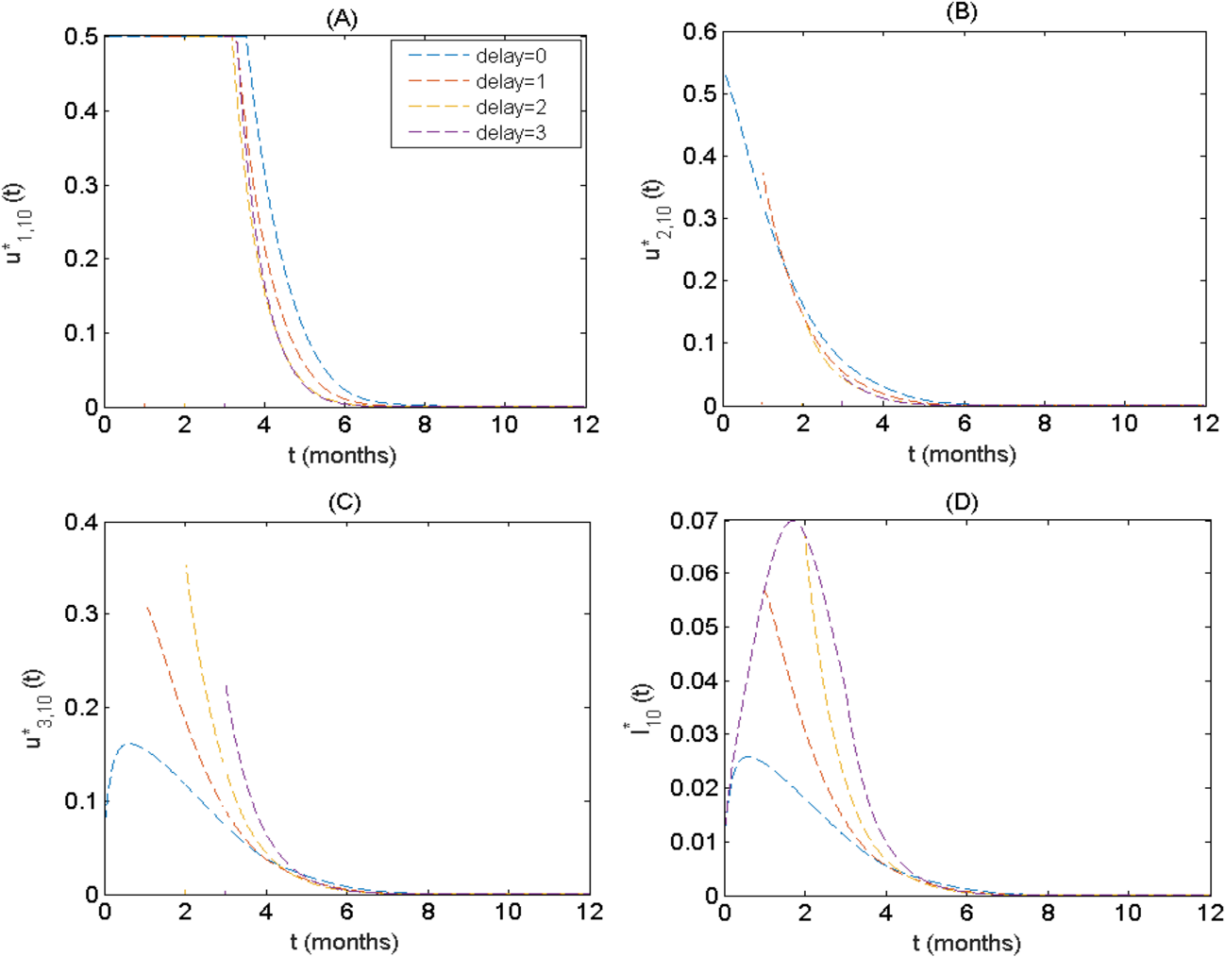
(**A**)–(**C**) The time series of optimal control function for group 10 (i.e. $k=10$) under four different timings of the implementation of control measures ($\mbox{delay time} =0,1,2,3$ month); (**D**) the corresponding densities of infected nodes in group 10

The timing of the implementation of interventions is always a critical issue. To do this, we incorporate a time delay in our model simulations by varying the start time of interventions: 1 month, 2 months, 3 months after the disease onset. The effect of time delay is evaluated in terms of optimal control functions, average control strength and densities of infected nodes among different groups. It is observed that the general shapes of control functions are similar except for the magnitudes among different groups, so we only illustrate the group 10 (i.e. $k=10$). Comparing the optimal control functions $u^{*}_{1,10}(t)$, $u^{*}_{2,10}(t)$, $u^{*}_{3,10}(t)$ under different starting times the control measures are carried out in Fig. 7(A)–(C), we see that as the time delay increases, the duration that the optimal control $u^{*}_{1,10}(t)$ being at the maximum level reduces while the initial intensity of optimal control $u^{*}_{2,10}(t)$ also decreases. In contrast, the initial intensity of optimal control $u_{3,10}^{*}(t)$ first increases and then declines with delaying the implementation of control strategies. Figure 7(D) displays that the delay in carrying out interventions leads to a significant increase in the densities of infected individuals. This indicates that the earlier the control measures is strengthened, the lower the outbreak size is.

**Figure 8:**
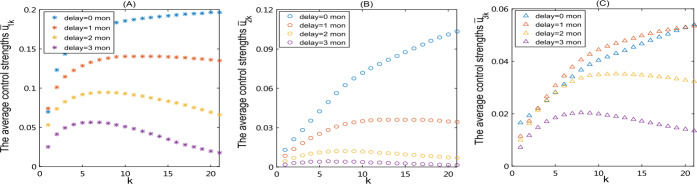
(**A**)–(**C**) Under the optimal controls, the average control strength of each group under four different starting times of controls ($\mbox{delay time} =0,1,2,3$ month)

Furthermore, we plot the average control strength $\bar{u}_{1k}$, $\bar{u}_{2k}$, $\bar{u}_{3k}$ under four different starting times of control measures. Figure 8 shows that as the starting time is delayed, the average control strength of interventions $\bar{u}_{1k}$, $\bar{u}_{2k}$ declines, especially for the group with high degree *k*. This is because the relatively late implementation of control measures leads to the disease spreading out and then susceptible individuals quickly declining. In particular, the subpopulation with great connectivity is infected faster. Hence, the highest intensity of control measures associated with personal protection and enhancement of vaccination is implemented in the groups with medium degrees rather than in the groups with high degrees. This means that with time delay increasing, the control intensity for groups with medium degrees should be relatively stronger, compared to other classes, to achieve more effective control.

#### Optimal control strategy on SF network

To investigate the effect of different network structures on the control of disease, we compare the main results on scale-free network with those on random network. In contrast to the random network, we find from Fig. 6 (B) that, for the SF network, the costs among total groups follows the exponential distribution and a substantially large fraction of the costs is occupied by the nodes with low degrees, which suggests in SF network, more resources should be allocated to these corresponding groups. This indicates that the distribution of costs among various groups coincides with the distribution of contact pattern. In the SF network, most nodes have low degrees, which yields great resource requirement, while in the random network, most nodes are those with medium degrees and hence they occupy most resources.

Numerical studies exhibit that the optimal control functions in the SF network behave like those in the random network, and particularly, the nodes with high degree require high intensity of interventions. The specific plots are like Fig. 4 and we omit them. Similarly, with the starting time of control measures being delayed, the optimal control intensity for total groups decreases, especially for the group with large *k*, while the corresponding densities of infected individuals increase. Again, the plots for the SF network are almost the same as Fig. 7 and Fig. 8, hence we omit them.

## Discussion

In recent years, the repeated outbreaks of domestic measles were of small-scale and clustered patterns, which presented obvious heterogeneity and attracted the public concern to design effective control measures against measles. To investigate the influence of heterogeneity on the measles transmission dynamics, we extend the homogeneous measles model to the heterogeneous network model. We further consider the waning of immunity in the proposed model and then divide the vaccinated individuals into two classes (fully protected with high antibody titer, probably infected with low antibody titer). We explore what control strategies should be implemented for individuals with different connectivities by using the optimal control method on the network model (high dimension). This network modeling approach provides a natural description to the transmission of measles among heterogeneous communities and we can obtain the detailed control scheme for individuals with different activities.

We initially analyze the threshold dynamics for the network model by defining the basic reproduction number $R_{0}$, which is the spectral radius of the next generation operator. We prove that the disease-free equilibrium $E_{0}$ is globally asymptomatically stable for $R_{0}<1$. While, if $R_{0}>1$, the disease-free equilibrium $E_{0}$ is unstable and the system is uniformly persistent. The basic reproduction number $R_{0}$ can act as a threshold to determine whether the disease dies out or not. Then we numerically study the effect of waning immunity and find the larger the waning rate is, the greater the basic reproduction number $R_{0}$ and the density of infected individuals are, which demonstrates that the long-term protection of vaccine is effective in controlling transmission of measles. We investigate the influence of network structure on $R_{0}$ for two kinds of networks: the SF network (with degree distribution $p(k)=mk^{-v}$) and the random network (with degree distribution $p(k)=\frac{K_{a}^{k}e^{-K_{a}}}{k!}$, $K_{a}=Nq$). Our results demonstrate that $R_{0}$ varies with the degree distribution for each network. The larger the parameter *v* or the smaller the parameter *q* of degree distribution is, the smaller $R_{0}$ is. This means we can effectively mitigate the outbreak of the disease by reducing the proportion of the highly connected individuals given contact structure in the case of the SF network, or restricting the contacts between nodes in the random network. Moreover, the comparison of $R_{0}$ between SF network and random network indicates that there exists a critical average degree $\bar{K}_{a}$ below which the basic reproduction number $R_{0}$ for the random network is larger than that, for the SF network, above which increasing the average degree induces a quick increase in $R_{0}$ (and thence new infections) for the SF network.

Furthermore, we extend the above network model by including three types of interventions (personal protection, enhancement of vaccination and treatment) to design the optimal control measures for achieving a low level of infections at a minimal cost. First, we prove the existence of the optimal solutions and characterize the optimal control functions using Pontryagin’s maximal principle. The numerical results indicate that, no matter what the network is, the outbreak peaks early with relatively large magnitude for the subpopulation with high activity, hence these individuals require a high intensity of interventions. With the optimal controls, the densities of infected nodes decrease by more than 50% for each group. Given delays of initiation of control measures, we see that late implementation of the interventions induces the relatively high density of infected individuals. This suggests that the control measures should be carried out as soon as possible. With the timing of implementation of control measures varying, the objective subpopulation implemented to the highest control strength also changes from individuals with high degrees to individuals with medium degrees, which is due to the delay of initiating controls results in the rapid spread of disease and further a rapid reduction of susceptible individuals, especially for those with great activities.

Our findings also demonstrate that, for the random network and the SF network, the costs occupied by different groups follow their own contact distribution. In particular, the distribution of costs is the normal distribution in the random network, while it follows the exponential distribution in the SF network. The results suggest us when the disease takes off in a community whose contact structure is more like random network, more resources should be allocated to the subpopulation with medium degrees due to most nodes are located in these groups. Conversely, in the SF network, most individuals have low degrees, so they should be paid more attention to and require more resources.

In summary, we developed a heterogeneous model with periodic transmission rate to investigate the effect of contact network and waning immunity on transmission of measles, which is an advantage compared to most existing homogeneous measles models. Furthermore, the effective control strategies for subpopulation with different activities are given by investigating the optimal control problem for the heterogeneous model. The results improve our understanding for the spread of measles and provide us with a detailed control scheme for different subpopulations, which cannot be obtained from the homogeneous models. However, it is challenging to obtain the realistic contact network due to the lack of reliable contact data, hence our simulations remain more qualitative. Moreover, it is essential to consider the effect of both age structure and contact heterogeneity on measles transmission, and we leave this for future work.

## Data Availability

Not applicable.
